# Pharmacist and Pharmacy Student Vaccination Authority: An International Survey of Pharmacy Educators

**DOI:** 10.3390/pharmacy14070150

**Published:** 2026-10-13

**Authors:** Kimberly McKeirnan, Ilse Truter, Teri-Lynne Fogarty

**Affiliations:** 1 Department of Pharmacy, Nelson Mandela University, Gqeberha 6031, South Africa; ilse.truter@mandela.ac.za (I.T.); teri-lynne.fogarty@mandela.ac.za (T.-L.F.); 2 Department of Pharmacology, Washington State University, Spokane, WA 99202, USA

**Keywords:** pharmacist scope of practice, vaccination authority, pharmacy student authority, regulatory variation, perceived benefits, qualitative content analysis

## Abstract

Background/Objectives: Pharmacists’ vaccination authority has expanded globally over the past four decades, but the scope of practice, meaning which vaccination activities pharmacists are legally authorised to perform, varies substantially by country. Pharmacy student authority to vaccinate, continuing education requirements, and pharmacy educators’ own perceptions of the benefits of pharmacist-administered vaccination have received comparatively little systematic, cross-national attention. This study aimed to describe pharmacist and pharmacy student scope of practice regarding vaccine administration, continuing education requirements, and pharmacy educators’ perceptions of the benefits of pharmacist-administered vaccination, across an international sample of countries. Methods: A cross-sectional survey of pharmacy educators was administered via Qualtrics between January and April 2026. Eighty-one responses representing 35 countries were analysed. Associations between pharmacist and student vaccination authority were tested using chi-square and Fisher’s exact tests, with Cramér’s V as the effect-size statistic, and the relationship between vaccination frequency and perceived benefits was assessed using Spearman’s correlation. Three open-ended items were analysed using inductive qualitative content analysis. Results: Most respondents (65.4%) reported that pharmacists in their country have conditional vaccination authority. Pharmacy students remained largely unauthorised overall (69.6% report no student authority), and even among institutions where pharmacists were authorised to vaccinate, most (57.1%) reported that students were not. Pharmacist and student authority were significantly associated (χ^2^ = 12.20, *p* < 0.001; Cramér’s V = 0.39), and perceived benefit correlated with how frequently pharmacists vaccinated (ρ = 0.44, *p* < 0.001). Restricted or unclear regulatory authority was the most frequently cited challenge, and a recurring theme across items. Pandemic-era vaccination authority showed mixed retention afterward, narrowing in some countries while persisting or expanding in others. Conclusions: Restricted or unclear regulatory authority was the most frequently reported barrier to expanding pharmacist- and pharmacy-student-led vaccination among respondents. Extending authority to appropriately trained pharmacy students and codifying pandemic-era emergency authority into permanent regulation are potential policy directions worth further investigation.

## 1. Introduction

Pharmacist-administered vaccination has evolved considerably since its earliest documented example in Argentina in 1979 [1]. The modern movement subsequently expanded practice in the United States during the 1980s and 1990s before spreading to Canada, Australia, the United Kingdom, and other countries [2,3,4]. Within the U.S. specifically, the scope of pharmacy-based immunisation statutes has varied considerably by state, with more permissive statutes associated with higher influenza vaccination rates [5].

This expansion has not been uniform. Scope of practice, meaning the services and professional activities pharmacists are legally authorised to perform, varies substantially by country. In some jurisdictions pharmacists may administer vaccines across the life course, while in others authority is limited to selected adult vaccines, seasonal influenza, COVID-19, travel vaccines, or emergency campaigns [1]. International Pharmaceutical Federation (FIP) surveillance data illustrate this variation. In a 2025 report, 56 out of 116 countries surveyed had legislation enabling pharmacy-based vaccination, up from 34 in 2020 [6]. In 43 of these 56 countries, pharmacists themselves were authorised to administer vaccines [6].

Authority to administer immunisations depends on further legislation, training requirements, reimbursement, access to vaccination records, and integration with immunisation systems [1]. Pharmacy students’ scope of practice, continuing education requirements for practising pharmacists, and the degree to which countries recognise pharmacist-administered vaccination as beneficial have received comparatively less systematic attention [7].

Where pharmacists have been granted vaccination authority, the reported benefits are substantial. Community pharmacies offer convenient locations, extended hours, and appointment-free access to care, reducing practical barriers to vaccination such as travel time, scheduling difficulty, and waiting times [8,9,10]. A systematic review and meta-analysis found that pharmacist involvement as an immuniser, vaccine advocate, or both was associated with improved immunisation uptake compared with usual care [11,12], and pharmacy-based vaccination services have been credited with improving accessibility for underserved populations [13,14]. Much of this evidence, however, comes from high-income countries and adult vaccination services, particularly influenza vaccination, limiting its transferability to other regions and to paediatric populations [11,14]. Evidence from low- and middle-income countries is comparatively limited [13] and largely comprises single-country surveys of pharmacy personnel’s readiness and willingness to vaccinate, such as a 2024 survey of 46 community pharmacy professionals in two Ethiopian towns, most of whom agreed that no regulation permitted them to do so [15]. Such studies rarely describe the regulatory position across countries or address pharmacy students.

Legal authorisation alone does not guarantee that pharmacist-administered vaccination is implemented consistently or well. Regulatory frameworks must also define standards for training, competency assessment, continuing professional development, and appropriate referral [4]. South Africa illustrates how such standards can be formalised: the South African Pharmacy Council has issued guidance describing scope of practice, competency standards, and accreditation criteria for pharmacist immunisation training [16,17], though this guidance is context-specific and should not be assumed to represent regulatory conditions elsewhere. Several regulatory barriers may limit pharmacist involvement in vaccination more broadly, including absence of explicit legal authority, unclear interpretation of existing pharmacy laws, restrictions based on patient age or vaccine type, and lack of integration with national immunisation programmes [18]. In FIP’s 2024 survey of 71 countries, limited government or health-system support was the most frequently reported barrier (48 countries), whereas limited access to training and low pharmacist confidence were reported less often [6]. Recent work has examined these barriers systematically, alongside facilitators such as training and stakeholder engagement [18], and has documented professional willingness to vaccinate persisting even where training and legal clarity remain limited [19].

Pharmacy students’ scope of practice presents a distinct regulatory question from pharmacist authority and one that has received comparatively little attention. Even where registered pharmacists hold established vaccination authority, pharmacy students do not automatically share in it. Authorisation to vaccinate as a student is typically governed separately and often lags. This distinction matters for workforce development. Students who are trained but not authorised to vaccinate lose the opportunity to utilise that training through supervised practice before entering the workforce [20]. Where standardised training and authority are absent altogether, the practical consequences can be significant. In Nigeria, the lack of standardised vaccination administration training in pharmacy curricula has been identified as a barrier limiting pharmacists’ capacity to contribute to national immunisation efforts, despite broad professional interest in expanding their role [21].

Existing international descriptions of pharmacist vaccination authority do not examine whether pharmacist and pharmacy student authority vary, whether perceived benefit is associated with vaccination frequency, or whether pandemic-era emergency authority has been retained or rescinded post-COVID-19. Empirical, educator-reported evidence testing these relationships across countries has not previously been documented. The purpose of this study was to evaluate and describe pharmacist and pharmacy student scope of practice regarding vaccine administration, continuing education requirements, and pharmacy educators’ perceptions of the benefits of pharmacist-administered vaccination globally.

## 2. Materials and Methods

### 2.1. Study Design and Companion Manuscript

This cross-sectional survey study describes pharmacist and pharmacy student scope of practice regarding vaccine administration, and pharmacy educators’ perceptions of the benefits associated with pharmacist-administered vaccination, across an international sample of countries. This study aimed to gather international perspectives from pharmacy educators rather than to achieve a statistically representative global mapping of vaccination authority. Data reported here were collected as part of a broader survey of pharmacy educators addressing both regulatory/practice conditions and vaccination curriculum content; findings related to curriculum content, training delivery, and educational adequacy are reported in a companion manuscript [McKeirnan et al., submitted to Pharmacy simultaneously with this manuscript]. The International Pharmaceutical Federation (FIP) maintains the World List of Pharmacy Schools as a publicly available, ongoing registry of pharmacy education institutions worldwide [22], which was used as the foundation for conducting the survey.

### 2.2. Instrument Development

Survey items addressing pharmacist and pharmacy student scope of practice, and perceived benefits of pharmacist-administered vaccination, were developed alongside the curriculum-focused items described in the companion manuscript, drawing on a prior systematic review of immunisation administration training for African pharmacists and student pharmacists [23] as well as published evidence on pharmacist-administered vaccination scope of practice and regulatory variation internationally described above [8,9,10,11,12,13,14]. The instrument was piloted with peers working in pharmacy vaccination-related education, resulting in an estimated completion time of approximately six minutes. The full instrument, administered via the Qualtrics Survey Platform, comprised nine blocks with built-in skip logic. Questions from the four blocks included in this article are shown in Table 1.

The authority items asked about “your country” and did not ask respondents to specify a state, province, or other jurisdiction. In countries where authority is regulated sub-nationally, responses therefore reflect the respondent’s understanding of the position in their own jurisdiction or a national summary, and these could not be distinguished.

### 2.3. Sampling Frame and Recruitment

The sampling frame underlying this recruitment was constructed from the International Pharmaceutical Federation’s (FIP) World List of Pharmacy Schools. At the time of access (January 2026), the list identified pharmacy schools across 146 countries. Every listed institution was targeted in countries with 20 or fewer schools. In countries with more than 20 schools (e.g., Brazil, the United States), a purposive subset of the top 20 institutions based on national ranking was identified using the AI-assisted search tool Microsoft Copilot version M365 (Microsoft Corporation, Redmond, WA, USA), prioritising representativeness within high-volume countries over exhaustive coverage that was not feasible within the scope of this study.

Contact identification followed a consistent tiered logic across targeted institutions. Microsoft Copilot was first used to locate the email address of a pharmacy school leader (dean, department chair or head, or director) engaged in pharmacy education or practice; where no such contact could be identified, an admissions email address was sought instead, on the reasoning that role-specific addresses are more likely to receive regular attention than general institutional inboxes, which were deliberately avoided based on the research team’s prior experience of low responsiveness. Where Copilot identified an institutional website but no specific contact, the corresponding email address was located manually. For institutions where no contact could be identified through either route, authors of published pharmacy education literature from the relevant country were identified and approached instead, reducing gaps in country coverage that would otherwise have resulted from limited institutional contact information. This combined approach yielded 823 recruitment emails representing all 146 countries with a listed school, constituting the full potential respondent pool for this study. Recipients unfamiliar with the vaccination-related scope of practice or regulatory conditions at their institution were invited to forward the survey to a better-positioned colleague, meaning the eventual respondent at a given institution may not always match the originally identified contact.

### 2.4. Data Collection

Recruitment was conducted electronically in sequential batches organised by continent between January and April 2026, with each batch remaining open for three weeks. Non-respondents received a single reminder email one week into each batch, restating the survey’s purpose and estimated completion time, offering the option to forward the survey to a better-placed colleague, and thanking those who had already responded. Recipients could also opt out of further recruitment contact at any point.

The survey was administered electronically using the Qualtrics Survey Platform (Qualtrics, Provo, UT, USA). Recruitment emails directed recipients to a survey link, which opened to a consent page prior to survey access. Recipients were invited to complete the survey themselves or forward it to a colleague more familiar with their institution’s vaccination-related practices.

### 2.5. Data Analysis

#### 2.5.1. Quantitative

Responses were analysed descriptively (frequencies, percentages) for all reported items. Item-level denominators vary due to skip logic and are reported alongside corresponding results. Since vaccination authority was assessed via respondent self-report rather than verified against national legislation, ‘authority’ as used throughout this manuscript refers to respondents’ reported understanding of their country’s regulatory position rather than an independently confirmed legal status. The association between pharmacist and pharmacy student vaccination authority was tested using the chi-square test of independence, with Fisher’s exact test reported alongside it given the categorical structure of the data, and Cramér’s V as the effect-size statistic.

Educators from countries with pharmacist vaccination authority rated four statements (Table 1) on a 5-point scale from 1 (strongly disagree) to 5 (strongly agree): that pharmacist-administered vaccination has increased the number of vaccinations given, increased access, reduced vaccine hesitancy, and reduced vaccine barriers. The items were developed for this survey from published evidence on the reported effects of pharmacist vaccination [8,9,10,11,12,13,14] to capture complementary aspects of a single construct, educators’ overall perception of benefit. They were combined because the sample was too small to model each outcome separately and the study’s question concerned overall perceived benefit. The four items were equally weighted and the score is the mean item rating rescaled to 0–100 [(mean − 1)/4 × 100], with higher values indicating stronger agreement. The rescaling aids interpretation only and does not affect the rank-based tests used. The items are not from a previously validated instrument, and with 56 respondents we could not assess factor structure, so the score provides only an exploratory summary of perceptions rather than a validated measure.

The relationship between vaccination frequency and the composite Benefits Score was assessed using Spearman’s rank correlation, and differences in Benefits Score between institutions with and without student vaccination authority were compared using the Mann–Whitney *U* test. A *p*-value less than 0.05 was considered statistically significant [24].

#### 2.5.2. Qualitative

Three open-text items invited respondents to describe successes, challenges, and other reflections regarding vaccination by pharmacists and pharmacy students in their countries. We analysed these responses using inductive qualitative content analysis [25,26]. This approach was chosen because the data were short, unprompted free-text entries embedded in a structured survey rather than interview or focus-group transcripts. The intent of the qualitative analysis was to describe the range and relative frequency of reported issues across countries rather than to develop an interpretive account of respondents’ comments, for which reflexive thematic analysis is better suited [27,28]. Since no established framework exists for classifying successes and barriers to pharmacist and student vaccination across countries, categories were derived from the data rather than imposed in advance.

Responses were exported from Qualtrics to Microsoft Excel (Microsoft Corporation, Redmond, WA, USA) and read in full several times. Each response was the unit of analysis, so within it, meaning units (words, phrases, or sentences expressing a distinct idea) were assigned open codes, using respondents’ own terms where possible. There was no pre-existing codebook. The code list was developed and revised during coding, and earlier responses were revisited when codes were refined. Each item was coded separately. Codes were compared and grouped into themes according to conceptual similarity, and each theme was given a name and working definition. Themes were not mutually exclusive, so a response was counted once under each theme it addressed. Country of origin was retained with each response so that patterns could be considered in a national context. One response consisting solely of an automated link was excluded.

The coder [K.M.] repeatedly returned to the raw responses to confirm that each code and theme fit the data. Two co-authors [I.T., T.-L.F.] reviewed all coded responses against the draft theme definitions independently. Disagreements were resolved by discussion and consensus. An audit trail of code lists, definitions, and decisions was maintained. Member checking was not possible because responses were anonymous. Verbatim quotes and countries are reported with each theme so readers can judge the fit between data and interpretation.

Responses that did not fit an emerging theme, or that expressed contrary views (for example, respondents who described existing vaccination systems as adequate without pharmacist involvement), were retained and examined, then used to refine theme boundaries or reported as separate themes. Where respondents from the same country described different circumstances, both accounts were retained.

The coder was a practising pharmacist and educator who helped design the survey, which may have influenced which concepts were noticed as salient. Given the small size and brief nature of the data (26–44 responses per item), findings are presented as illustrative and exploratory. They describe the range of views reported and are not exhaustive or generalisable. Theme counts show how often a topic was raised within this sample and should not be read as prevalence; the absence of a theme does not mean other respondents disagreed with it. Reporting followed applicable items of the SRQR [29].

### 2.6. Ethical Approval

This study was approved by the Research Ethics Committee (Human) at Nelson Mandela University (reference number H23-HEA-PHA-007) prior to the commencement of data collection. The study was conducted in accordance with the ethical principles of respect for persons, beneficence, and justice, consistent with the Belmont Report (National Commission for the Protection of Human Subjects of Biomedical and Behavioural Research, 1979) [30].

Participation was entirely voluntary. Potential participants were informed, via a consent page presented before survey access, of the study’s purpose, the researchers’ institutional affiliation, and their right to decline participation, skip individual items, or withdraw at any time without consequence. Only respondents who explicitly indicated consent were permitted to proceed to the survey; those who declined were directed to a page thanking them for their time, and no further data were collected from them.

Responses were collected anonymously through the Qualtrics Survey Platform. All data were collected anonymously, including only information that was provided by the respondents, and were stored on password-protected devices accessible only to the research team, and results are reported in aggregate throughout this manuscript. No individual respondent or institution is identifiable in any reported finding.

Given the international scope of recruitment, the researchers also confirmed that no additional institutional ethics review was required beyond the approval granted by Nelson Mandela University’s Research Ethics Committee.

## 3. Results

### 3.1. Response Rate and Respondent Characteristics

Of the 823 recruitment emails distributed, 108 responses were received (13.1%). Following the exclusion of one non-consenting respondent, 25 records with no substantive content beyond country and institution, and one test entry, 81 completed responses remained, representing a final response rate of 9.8%.

Respondents represented pharmacy educators from 35 countries (24.0%) spanning all six FIP regions, most heavily from Europe (12 countries) and Africa (10 countries), with smaller representation from the Eastern Mediterranean (4 countries), the Western Pacific (4 countries), the Americas (3 countries), and South-East Asia (2 countries). Across the 81 respondents who reported their country of employment, 79 identified a specific institution. Among those who reported gender (*n* = 64), a slight majority identified as female (53.1%). Institutional length of employment varied considerably: among the 67 respondents who reported years at their current institution, over half (53.7%) had worked there for more than 10 years, while a small minority (1.5%) had been at their institution for less than one year.

### 3.2. Pharmacist Vaccination Authority, Training, and Continuing Education

Most respondents (65.4%) reported that pharmacists in their country have conditional vaccination authority, while 28.4% reported no authority and 6.2% reported unrestricted authority. Training programmes and continuing education were reported as readily available by most respondents, though roughly a third reported that updated training is not required to maintain approval to vaccinate. Results are shown in Table 2.

### 3.3. Perceived Benefits of Pharmacist-Administered Vaccination

Educators most strongly agreed that pharmacist vaccination had increased access to vaccinations (80.4% agree/strongly agree), while agreement was weakest for reduced vaccine hesitancy, where half of respondents were neutral, as shown in Table 3.

The four benefit items were combined into a composite Benefits Score (range 0–100), which had a mean of 72.8 (median 75.0, standard deviation 19.6).

### 3.4. Pharmacy Student Vaccination Authority and Training

Pharmacy student vaccination authority lagged considerably behind pharmacist authority, as shown in Table 4. Most respondents (69.6%) reported that students have no vaccination authority at all, compared with 28.4% who reported the same for pharmacists.

### 3.5. Association Between Pharmacist and Student Authority

Among the 56 respondents whose country grants pharmacists any vaccination authority, only 42.9% reported that students share in that authority, while 57.1% reported students have none. This reported association between pharmacist and student authority was significant and moderate-to-large (χ^2^ = 12.20, df = 1, *p* < 0.001; Fisher’s exact *p* < 0.001; Cramér’s V = 0.39).

### 3.6. Benefits Score, Vaccination Frequency, and Student Authority

The composite Benefits Score correlated significantly and moderately with how often pharmacists report vaccinating (Spearman’s ρ = 0.44, *p* < 0.001, n = 56). Educators in countries where pharmacist-administered vaccination is more routine perceived greater benefit. Since this association is correlational, the direction of this relationship cannot be established from these data; greater perceived benefit may encourage more frequent practice, or more frequent practice may itself generate more visible, perceived benefit.

Median Benefits Score was somewhat higher in countries where pharmacy students also have vaccination authority (81.25) than where they do not (75.0), though this difference did not reach statistical significance (Mann–Whitney *U* = 271.0, *p* = 0.060) and is best interpreted as a trend rather than a confirmed effect.

The following three sections present findings from the survey’s three open-ended items, analysed using inductive qualitative content analysis as described in Section 2.5.2.

### 3.7. Successes Reported by Respondents

Increased access to vaccination services, particularly for underserved populations, was the most frequently reported success theme, though a substantial minority of respondents reported no success to report at all, since pharmacist-administered vaccination was not yet an option in their country. Themes and illustrative quotes are shown in Table 5.

### 3.8. Challenges Reported by Respondents

Restricted or unclear regulatory authority was the most frequently reported challenge, cited more than twice as often as any other theme, as shown in Table 6. Notably, this barrier applied not only to pharmacists broadly but also, in a distinct subset of responses, specifically to pharmacy students in countries where registered pharmacists already hold vaccination authority.

Restricted or unclear regulatory authority for pharmacists and pharmacy students was the most consistently reported theme, recurring within both the challenges and “other” items. It also recurred within the successes item as an explicit absence.

### 3.9. Other Reflections Reported by Respondents

Respondents’ additional reflections most often described incremental progress: countries actively building or expanding training capacity or acknowledging genuine progress alongside work still to be done, together accounted for over a third of responses in this category. These reflections are shown in Table 7. 

Theme counts do not sum exactly to the total *n* for each item, as a small number of responses addressed more than one theme and were counted in each, while a small number of singleton themes are omitted from the tables for brevity.

## 4. Discussion

This study was conducted because, despite pharmacist vaccination authority expanding across many countries over the past four decades, scope of practice remains highly heterogeneous: some countries permit unrestricted, life-course vaccination by pharmacists, while others restrict authority to specific vaccines, patient populations, or emergency circumstances, with authority shaped by each country’s distinct legislative, professional, and health-system context [1]. Pharmacy student authority, continuing education requirements, and educators’ own perceptions of the benefits of pharmacist-administered vaccination had received comparatively little systematic, cross-national attention prior to this study [7]. The findings reported here suggest that this variation may correspond to the relationship between pharmacist and pharmacy student authority, and to a set of barriers that educators themselves consistently identify as regulatory rather than educational in nature.

### 4.1. Pharmacist and Student Authority Were Associated, and Perceived Benefit Was Associated with Vaccination Frequency

Prior evidence suggests that the scope of practice can remain inconsistent even in countries with long-established pharmacist vaccination authority. In the United States, despite pharmacists gaining vaccination authority in all 50 states by 2009 and administering the majority of COVID-19 and adult vaccines nationally, state-to-state variation in immunising authority by patient age and vaccine type, along with inconsistent payer reimbursement, continues to present challenges [31]. This state-to-state statutory variation has measurable consequences: a national analysis found that the scope of pharmacy-based immunisation statutes was associated with influenza vaccination rates, with more permissive statutes corresponding to higher uptake [5]. This study’s own findings suggest a similar pattern operates globally and point to two related quantitative results. First, reported pharmacist and pharmacy student vaccination authority were significantly associated (χ^2^ = 12.20, *p* < 0.001; Cramér’s V = 0.39): in countries where pharmacists were reported to be able to vaccinate, students were more likely to be able to vaccinate as well when compared with countries where pharmacists could not vaccinate. Second, perceived benefit aligned with how often pharmacists vaccinated (Spearman’s ρ = 0.44, *p* < 0.001), suggesting that visible practice, rather than policy alone, may be associated with educator confidence in pharmacist-administered vaccination. This aligns with prior evidence that pharmacist-administered vaccination services have measurably improved access in underserved communities specifically [32], lending external support to educators’ strong agreement that pharmacist-administered vaccination increases access. A parallel, though not statistically confirmed, pattern emerged for student authority: composite Benefits Score was somewhat higher where students also held vaccination authority, though this difference fell just short of significance (Mann–Whitney *U* = 271.0, *p* = 0.060). Together, these findings suggest that expanding student authority specifically, not only pharmacist authority, may be associated with more favourable perceptions of pharmacist-administered vaccination overall.

### 4.2. Regulatory Authority, Not Professional Readiness, Was Reported as the Primary Barrier

The qualitative findings are consistent with the quantitative patterns. Across all three open-ended items, restricted or unclear regulatory authority was the most consistently reported theme, appearing as an explicit absence within the successes item (“we haven’t succeeded to get [sic] the permission yet,” Türkiye) and as the leading challenge theme overall. A more specific pattern within this broader authority gap was also identified: several respondents, concentrated heavily in Australia, described a situation in which registered pharmacists hold vaccination authority, but pharmacy students do not, even after completing formal training. One Australian respondent noted that trained students remained barred from vaccinating even under supervision, and a South African respondent attributed the gap explicitly to institutional bureaucracy [sic] rather than any deficit in student competency (“the only challenge is this bureaucracy where now our students can’t [sic] vaccinate despite being well trained”). This is a materially different problem from a general absence of pharmacist authority: it may limit experiential learning opportunities and delay workforce readiness, since students who might otherwise graduate fully prepared to vaccinate must instead wait until after registration to gain hands-on experience. Expanding vaccination authority to appropriately trained pharmacy students, rather than to registered pharmacists alone, may warrant consideration in jurisdictions where pharmacist authority is already established.

This pattern echoes findings from outside this survey’s own respondent countries. In Nigeria, despite growing training infrastructure and a national survey finding that 91% of community pharmacists expressed interest in administering vaccines, the absence of a clear legal and regulatory framework has continued to limit formal recognition of pharmacists’ vaccination role [21,33], a pattern also documented among community pharmacists in Cross River State, Nigeria, where participation in immunisation services was similarly constrained more by regulatory and systemic factors than by professional willingness [34].

The qualitative data also show that a single national answer can mask sub-national differences. Respondents from Canada and Australia described province- or state-level variation in what pharmacists may administer. This parallels the state-based variation long documented in the United States [31] and reported by FIP in Australia, where differences between states affect which vaccines pharmacies can administer, and in Italy and Finland, where regional differences produce uneven access [6]. Such fragmentation may contribute to inconsistent training and practice, and FIP notes that harmonisation efforts are under way in Canada and Australia [6,20] and recommends harmonising adult vaccination policies across jurisdictions, particularly in federated or decentralised systems [20]. Because our survey asked about “your country” and we cannot say how respondents in these settings interpreted the question, we treat our authority categories as educator-reported summaries and not as a legal classification.

### 4.3. Progress Achieved During the COVID-19 Pandemic Has Mixed Retention

A further pattern worth highlighting is the mixed retention of vaccination authority gained during the COVID-19 pandemic. Respondents from Jordan and the Netherlands independently described pandemic-era vaccination roles that were not sustained afterward, narrowing back to a limited vaccine set in Jordan’s case or stalling altogether in the Netherlands. Elsewhere, pandemic-driven expansion has persisted: in South Africa, the pandemic prompted efforts to empower practising pharmacists to vaccinate that appear to have endured [35,36], and in the United States, the Public Readiness and Emergency Preparedness (PREP) Act provided the legal basis for a pandemic-era expansion of vaccinator authority that similarly remained in place [37]. This divergence in post-pandemic retention suggests that emergency authority may function as a temporary rather than durable expansion of scope unless deliberately codified into permanent regulation, a distinction with direct relevance for policymakers considering how to convert crisis-driven flexibility into lasting practice change.

### 4.4. Signs of Active Progress, and Where Work Remains

Despite these barriers, the qualitative data show substantial forward movement already underway. New or expanded training programmes were described independently in Botswana, Cambodia, Eswatini, Georgia, and the UK, and several respondents explicitly framed their country’s position as one of genuine progress with acknowledged work still to do (Cyprus, India, Ireland, Kenya, Türkiye). Active capacity-building alongside a persistent gap between training and legal authority to practise suggests that the primary obstacle to expanding pharmacist- and pharmacy-student-led vaccination internationally may be regulatory rather than educational. Respondents report that many of the systems needed to train a vaccination-ready workforce are already being built, but the authority to deploy that workforce, particularly among pharmacy students, has not kept pace.

The barriers to this expansion are not solely regulatory, however. Opposition from other stakeholders, raised by respondents in Malaysia, Türkiye, and the UAE, is also documented internationally. Similar to our qualitative findings, FIP describes concerns among physicians and nurses about clinical oversight, patient safety, competition, and pharmacists’ training, and reports that resistance from medical stakeholders has slowed progress in Serbia and Türkiye [6,38]. Funding and financing constraints, including the cost of vaccine storage infrastructure, were raised independently by respondents in Australia and Ghana. A recent measles outbreak in Indonesia was cited by a respondent as illustrating the public health cost of insufficient vaccine education. This serves as a reminder that the case for expanding pharmacist- and pharmacy-student-led vaccination is epidemiological as well as professional.

### 4.5. Limitations

This study shares several limitations with its companion manuscript, which draws on the same respondent pool. Of 823 recruitment emails distributed, 81 usable responses were received, corresponding to a completed-response rate of 9.8%. Although low response rates are a frequent challenge with survey-based research, these results also limit the generalizability of the findings. The results reported here also reflect a self-selected subset of contacted institutions rather than a representative sample of global pharmacy education generally. Additionally, because recipients unfamiliar with their institution’s vaccination policies were invited to forward the survey, the respondent for a given institution was not always the originally identified contact, which may introduce inconsistency in respondent role or seniority across countries. Country-level coverage was uneven, with most of the 35 represented countries reflected by only one or two respondents. All data were self-reported, without independent verification against regulatory documentation, and the survey instrument was administered in English only, which may have limited participation from non-English-speaking regions.

There are also limitations with the quantitative analysis. The sample is relatively small and responses may not be independent if multiple participants came from the same country or institution. The Benefits Score combines four newly developed items that have not been formally validated, so the 0–100 range should not be read as implying precise measurement. In addition, the score reflects educators’ perceptions rather than measured outcomes. Both the score and vaccination frequency were reported by the same respondent, which may inflate their association through shared-source bias.

For the qualitative component specifically, coding was conducted by the first author/principal investigator, who has 18 years of experience in pharmacy vaccination practice and education. This familiarity may have shaped which concepts were noticed as salient during coding. No claim of thematic saturation is made, and formal inter-rater reliability statistics are not reported, consistent with the small, embedded nature of this qualitative component. Trustworthiness was instead supported through the retention of verbatim illustrative quotes for each theme, allowing readers to evaluate the fit between raw data and theme assignment directly. A thorough review of coded themes by both supervising co-authors (I.T. and T.-L.F.), who provided feedback on theme definitions and category boundaries prior to finalisation, also improves the quality of the results.

## 5. Conclusions

Pharmacist and pharmacy student vaccination authority are significantly associated. Restricted or unclear regulatory authority, rather than gaps in professional readiness, was the barrier most consistently reported by respondents. Expanding authority to appropriately trained pharmacy students and codifying pandemic-era emergency authority into permanent regulation may represent potentially feasible policy directions in jurisdictions where pharmacist authority is already established.

## Figures and Tables

**Table 1:** Survey items reported in this manuscript, by block.

Block	Item	Response Format
Demographics	In which country is the school or institution where you are employed located? If you teach/work at more than one institution, then please identify the country in which you teach/work most frequently.	Open text
	What is the name of the institutions where you are employed? As a reminder, individual responses and names of institutions will not be shared.	Open text
	How many years have you been working at your current institution?	4-category (Less than 1 year/1 to 5 years/6 to 10 years/More than 10 years)
	Which gender do you most identify with?	4-category (Male/Female/Non-binary/third gender/Prefer not to say)
Pharmacist Block	Are registered pharmacists in your country allowed to administer vaccinations as part of their scope of practice?	3-category (Yes, all can vaccinate without restrictions/Yes, some can vaccinate but only if they meet certain criteria [e.g., training, certification] or under certain circumstances, such as during the pandemic/No—skip to end of block if No)
	How frequently do pharmacists who are authorised to vaccinate in your country administer vaccinations?	5-category (Very often, it is a routine part of practice/Regularly, but with limitations/Occasionally, some pharmacists do this, but it is not common/Rarely, it is unusual for pharmacists to vaccinate/Never)
	Do pharmacists in your country who want to become trained to vaccinate have training programmes readily available to them?	Yes/No/Not sure
	Are pharmacists in your country required to complete vaccination-related continuing professional development (CPD) and/or continuing education (CE) for ongoing licensure?	Yes/No/Not sure
	Is vaccination-related continuing professional development (CPD) and/or continuing education (CE) readily available for pharmacists in your country?	Yes/No/Not sure
	Are pharmacists in your country who are trained to vaccinate required to take updated training courses to maintain approval to vaccinate?	Yes/No/Not sure
	Please rate your level of agreement: Having pharmacists administering vaccinations: has increased the number of vaccinations given in my country has increased access to vaccinations for people in my country has reduced vaccine hesitancy among people in my country has reduced vaccine barriers for people in my country	5-point Likert scale from 1 (Strongly Disagree) to 5 (Strongly Agree)
Pharmacy Student Block	Are pharmacy students in your country allowed to administer vaccinations as part of their scope of practice?	3-category (Yes, all can vaccinate without restrictions/Yes, some can vaccinate but only if they meet certain criteria [i.e., training, certification, etc.] or under certain circumstances [i.e., pandemic]/No—skip to end of block if No)
	How frequently do pharmacy students in your country administer vaccinations?	5-category (Very often/Regularly/Occasionally/Rarely/Never)
	Do pharmacy students in your country who want to become trained to vaccinate have training programmes readily available to them?	Yes/No/Not sure
Open-ended reflections	Please use the text box below to write in any information you would like to share with us about the success of having pharmacists, pharmacy students, and pharmacy support personnel vaccinating in your country.	Open text
	Please use the text box below to write in any information you would like to share with us about challenges or barriers to having pharmacists, pharmacy students, and pharmacy support personnel vaccinating in your country.	Open text
	Is there anything else you would like to share regarding vaccinations or vaccination training in your country?	Open text

Abbreviations used: CPD = continuing professional development; CE = continuing education.

**Table 2:** Survey responses regarding pharmacist vaccination authority, training, and continuing education n.

Survey Item	Category	n (%)
Pharmacist vaccination authority (*n* = 81)	None	23 (28.4%)
	Some, approval conditional	53 (65.4%)
	Approved, unrestricted	5 (6.2%)
Frequency of pharmacist vaccination * (*n* = 56)	Rarely	9 (16.1%)
	Occasionally	14 (25.0%)
	Regularly, but with limits	7 (12.5%)
	Very often	26 (46.4%)
Training programmes readily available * (*n* = 53)	Yes	44 (83.0%)
	No	9 (17.0%)
CPD/CE required for licensure * (*n* = 49)	Yes	32 (65.3%)
	No	17 (34.7%)
CPD/CE readily available * (*n* = 49)	Yes	37 (75.5%)
	No	12 (24.5%)
Updated training required to maintain approval * (*n* = 42)	Yes	22 (52.4%)
	No	20 (47.6%)

* Skip-logic subsample: reported only by respondents whose country grants pharmacists some form of vaccination authority.

**Table 3:** Perceived benefits of pharmacist-administered vaccination (*n* = 56).

Benefit Statement	Disagree/Strongly Disagree	Neutral	Agree/Strongly Agree
	n (%)	n (%)	n (%)
Increased vaccinations given	4 (7.1%)	14 (25.0%)	38 (67.9%)
Increased access	4 (7.1%)	7 (12.5%)	45 (80.4%)
Reduced vaccine hesitancy	6 (10.7%)	28 (50.0%)	22 (39.3%)
Reduced vaccine barriers	4 (7.1%)	12 (21.4%)	40 (71.4%)

**Table 4:** Survey responses regarding pharmacy student vaccination authority and training.

Item	Category	n (%)
Student vaccination authority (*n* = 79)	None	55 (69.6%)
	Occasional approval	10 (43.5%)
	Some approval, conditional	23 (29.1%)
	Approved, unrestricted	1 (1.3%)
Frequency of student vaccination * (*n* = 23)	Never	1 (4.3%)
	Rarely	6 (26.1%)
	Regularly, but with limits	4 (17.4%)
	Very often	2 (8.7%)
Training programmes readily available * (*n* = 21)	Yes	19 (90.5%)
	No	2 (9.5%)

* Skip-logic subsample: reported only by respondents whose country grants pharmacy students some form of vaccination authority.

**Table 5:** Successes reported by respondents (*n* = 39 *).

Theme	n	Countries Represented	Illustrative Quote
Allowing pharmacists to vaccinate has increased access, including reaching underserved populations.	12	Australia, Canada, Eswatini, Ireland, Malta, New Zealand, UK	“Delivering millions of doses and significantly boosting accessibility in deprived areas” (UK)
They have no success to report yet.	8	Botswana, Georgia, Ghana, India, Lebanon, Netherlands, Türkiye	“We haven’t succeeded to get the permission yet” (Türkiye)
New vaccination training programmes have been established.	6	Australia, Ghana, India, Kenya, Netherlands, UAE	“Other institutions have successfully trained practicing Pharmacists in vaccination… e.g., University of Nairobi” (Kenya)
New vaccination authority has been granted.	4	Australia, Cambodia, Ireland, Malaysia	“We got a letter from the MOH Malaysia that stated certified pharmacists may administer COVID-19 vaccines” (Malaysia)
Students are actively involved in vaccination delivery.	3	Canada, South Africa, UK	“Our students are trained in the fall of their first year and are able to inject under supervision… They play a large role in seasonal influenza campaigns” (Canada)

* Not all quotes represented, particularly when reported by only one participant.

**Table 6:** Challenges reported by respondents (*n* = 44 *).

Theme	n	Countries Represented	Illustrative Quote
Absent or unclear regulatory approval allowing pharmacists to vaccinate	20	Botswana, Cambodia, Cyprus, Georgia, Ghana, India, Indonesia, Jordan, Kenya, Lebanon, Malaysia, Norway, Türkiye, Yemen, UAE	“Lack of strong guideline and approvals from the authorizing bodies in my country to allow pharmacists to vaccinate various types of vaccines” (Jordan) “The barriers of pharmacist in my county is pharmacist is not allowed to inject or medication administration” (Cambodia)
No authority for students specifically, even where pharmacists can vaccinate	8	Australia, Ireland, New Zealand, South Africa	“The only challenge is this bureaucracy where now our students can’t vaccinate despite being well trained” (South Africa)
Curriculum and training not integrated or insufficient	6	Australia, Eswatini, Ghana, India, UK	“Lack of curriculum content and skills training on vaccination” (Ghana)
Opposition or lack of support from other stakeholders	3	Malaysia, Türkiye, UAE	“The Ministry of Health believes that we have a very successful vaccination programme in Türkiye which they do not need pharmacists to be involved [in]”
Workforce pressure, staffing shortages, and workload burden	3	UK, Canada	“The main hurdles are extreme workload pressures and staffing shortages” (UK)
Fragmented or inconsistent regulation across jurisdictions	2	Australia, Canada	“Limitations on what they can administer is State-based and so working across states can be challenging” (Australia)

* Not all quotes represented, particularly when reported by only one participant.

**Table 7:** Other reflections reported by respondents (*n* = 26 *^).

Theme	n	Countries Represented	Illustrative Quote
Positive progress has been made, but work remains	5	Cyprus, India, Ireland, Kenya, Türkiye	“Vaccination policies and practices in Türkiye are well-established and contribute effectively to public health, [but] there is still room for further improvement”
Training capacity has expanded or has the potential to be expanded	5	Botswana, Cambodia, Eswatini, Georgia, UK	“There is potential to expand the role of pharmacists in vaccination in Georgia by introducing targeted training programmes and updating regulations”
Persisting restrictions exist despite readiness and training	5	Cambodia, Lebanon, Malaysia, South Africa, UAE	“We have a good vaccination system in Lebanon, but the administration of vaccines is exclusively under the supervision of doctors”
Jurisdictional variation complicates implementation	3	Australia, Canada	“Every province in Canada has different regulations and rules regarding who can administer vaccines”
Positive momentum toward change exists	3	Malaysia, Norway, UK	“There is a positivism now to let pharmacists partake in this health care service” (Norway)
Funding and financing issues limit implementation	2	Australia, Ghana	“Financing is also a problem. Acquisition of storage facility etc.” (Ghana)
A public health need exists for pharmacist vaccination services	2	Ghana, Indonesia	“The measles outbreak in Indonesia recently have demonstrated the lack of vaccine education among Indonesian population”
Pharmacists are trained and ready but not authorised to vaccinate	2	India, Jordan	“This is only a part of the immunization subject curriculum in teaching, but practice is not allowed in our country for pharmacists”

* Not all quotes represented, particularly when reported by only one participant. ^ One invalid response excluded.

## Data Availability

The data presented in this study are available on request from the corresponding author, as the datasets presented in this article are not readily available due to restrictions from the Research Ethics Committee (Human).

## Abbreviations

The following abbreviations are used in this manuscript:

CPD	Continuing Professional Development
CE	Continuing Education
COVID-19	Coronavirus Disease 2019
FIP	International Pharmaceutical Federation
UK	United Kingdom
